# Protocol for human evaluation of generative artificial intelligence chatbots in clinical consultations

**DOI:** 10.1371/journal.pone.0300487

**Published:** 2025-03-19

**Authors:** Edwin Kwan-Yeung Chiu, Tom Wai-Hin Chung

**Affiliations:** Department of Microbiology, Li Ka Shing Faculty of Medicine, The University of Hong Kong, Hong Kong, China; Macao Polytechnic University, MACAO

## Abstract

**Background:**

Generative artificial intelligence (GenAI) has the potential to revolutionise healthcare delivery. The nuances of real-life clinical practice and complex clinical environments demand a rigorous, evidence-based approach to ensure safe and effective deployment of AI.

**Methods:**

We present a protocol for the systematic evaluation of large language models (LLMs) as GenAI chatbots within the context of clinical microbiology and infectious diseases clinical consultations. We aim to critically assess recommendations produced by four leading GenAI models, including Claude 2, Gemini Pro, GPT-4.0, and a GPT-4.0-based custom AI chatbot.

**Discussion:**

A standardised, healthcare-specific, universal prompt template is developed to elicit clinically impactful AI responses. Generated responses will be graded by two panels of practicing clinicians, encompassing a wide spectrum of domain expertise in clinical microbiology and virology, as well as infectious diseases. Evaluations will be performed using a 5-point Likert scale across four clinical domains: factual consistency, comprehensiveness, coherence, and medical harmfulness. Our study will offer insights into the feasibility, limitations, and boundaries of GenAI in clinical consultations, providing guidance for future research and clinical implementation. Ethical guidelines and safety guardrails should be developed to uphold patient safety and clinical standards.

## Introduction

With an aging global population, ever increasing healthcare demands and the rapid evolution of healthcare technologies, effective integration of artificial intelligence (AI) into clinical workflow and decision-making processes have become a focal point of research and debate. GenAI has demonstrated significant potentials in understanding natural language and addressing cognitive tasks. [1] The prospects of GenAI replacing or augmenting physician tasks, particularly in telemedicine where information exchange is primarily text-based, has prompted investigations into their practicality and safety in clinical consultations. [2]

Preliminary investigations have demonstrated the potentials for AI in managing various infectious disease syndromes (e.g., bloodstream infections and brain abscesses), however, concerns remain about the reliability, safety, and ethics of GenAI adoption in clinical practices.[3–5] This study is among the first to systemically evaluate state-of-the-art GenAI large language model (LLM) chatbots, including a custom AI chatbot (custom bot) integrated with domain-specific medical literature. In addition, this study employs a novel self-developed healthcare-specific prompt template purposely designed to examine AI chatbot performances in complex real-life clinical scenarios. A unique dual-tier evaluation system that includes both practicing specialists and non-specialist resident trainees is also implemented in the evaluation process to offer a comprehensive assessment from multiple levels of domain expertise and clinical experience.

The objective of this protocol is to critically assess the clinical accuracy, coherence, comprehensiveness, and safety of recommendations provided by AI chatbots. This research aims to contribute to the ongoing discourse on the role of GenAI in healthcare and to aid in the development of guidelines to ensure the safe and effective deployment of GenAI in clinical microbiology and infectious diseases.

## Materials and methods

**Fig 1:**
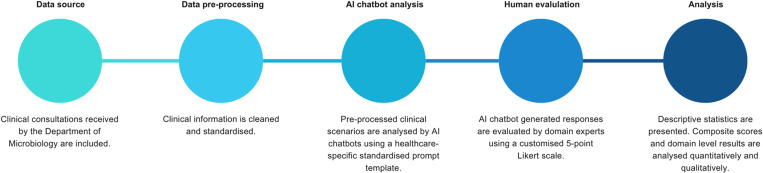
Materials and methods. AI: artificial intelligence.

This project aims to evaluate the potential role of AI chatbots to assist clinicians by providing immediate analysis and suggestions to enhance and augment clinical practice and workflow. The protocol employs a universal standardised prompt template to compare between AI chatbot responses against real-life clinical scenarios. Generated responses will be evaluated by a panel of practicing clinicians [specialists (n =  3); resident trainees (n =  3)] using a 5-point Likert scale. [6] Human evaluators will serve as domain experts with specific knowledge in clinical microbiology and virology, as well as internal medicine and clinical infectious diseases (Fig 1).

### Data source

During the pre-defined study period, real-life clinical notes will be extracted retrospectively from the digital depository (in-house software) of the Department of Microbiology, Queen Mary Hospital (QMH), Hospital Authority, Hong Kong. Within the study period, 40 clinical notes derived from four clinical microbiologists [10 clinical notes per microbiologists; fellows (n =  2) and resident trainees (n =  2)] will be included consecutively.

For the inclusion criterion, only new in-patient consultation referrals received by the Department of Microbiology (QMH) during the study period will be included. As for exclusion criteria, duplicated consultations will be removed to limit redundancy and potential data skew. Follow-up assessments and reviews during the same clinical episode will be excluded to focus on initial management approach, diagnostic assessments, and treatment decisions. The inclusion and exclusion criteria are carefully designed to maintain clarity and data integrity and to ensure a well-defined analytical framework.

### Data preprocessing

Data preprocessing will be conducted manually by E.K.Y.C and T.W.H.C. To maintain authenticity of the original clinical notes, preprocessing procedures are designed to be minimal, where the clinical context, syntax and written styles of the initial documents are retained as far as possible. Patient identifiable information is removed. Names of medical institutions are excluded or anonymised. Medical terminologies are standardised, where abbreviations and non-universal short forms are converted into their full terms (e.g., expanded abbreviations: from ‘c/st’ to ‘culture’, ‘T/F’ to ‘pending results’, ‘CMV D+R-’ to ‘cytomegalovirus seropositive donor and seronegative recipient’). Appropriate International System (SI) of units are included for quantitative results to allow clear interpretations (e.g., adding ‘g/dL’ to the values of haemoglobin). For chronological structuring, relevant dates are included in the clinical scenarios. Moreover, contents related to the clinical impression and management approach of the scenario will be removed to minimise input biases. Lastly, to ensure structural uniformity across all clinical scenarios, the contents will be outlined systematically into five categories: “Basic demographics & Underlying medical conditions”, “Current admission”, “Physical examination findings”, “Investigation results” and “Antimicrobials & Treatments”.

### Prompt template

**Fig 2:**
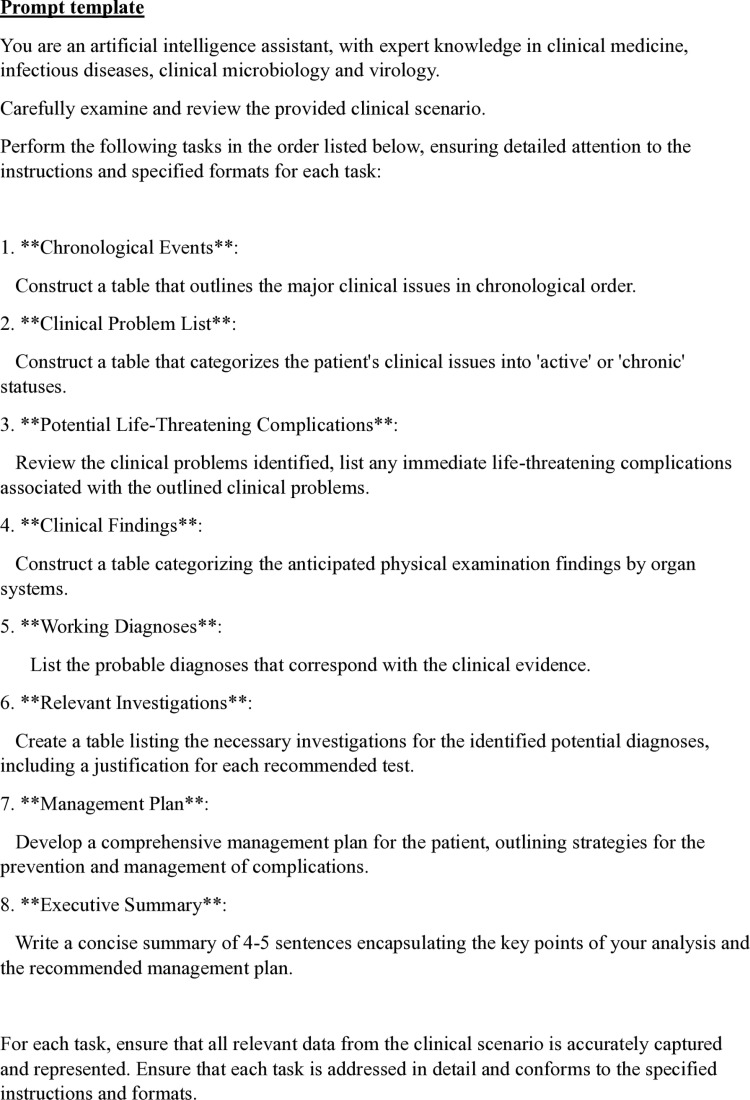
Healthcare-specific standardised prompt template.

A standardised, domain-specific, unconditional, zero-shot prompt template was developed for this study (Fig 2). All clinical scenarios will be processed as separate files along with the standardised prompt template. [7]

The prompt template begins with a system message (base prompt) that defines the behaviour of the model and prescribes the style of response within pre-defined boundaries. In this study, AI chatbots are primed as “an artificial intelligence assistant with expert knowledge in clinical medicine, infectious disease, clinical microbiology and virology”.

In the design of the prompt template, a Performed-Chain of Thought (P-COT) prompting approach will be adopted. [8] The analytical process will be broken down into clinically meaningful segments and sub-tasks, to allow a logical sequence of prompts, where the outputs permeate sequentially throughout the step-by-step process. [9, 10]

Initially, the chatbot is instructed to identify and classify active clinical problems, and list any immediate life-threatening complications, ensuring that each complication logically stems from the reviewed issues. Following this, the chatbot suggests anticipated physical examination findings which correlate to the identified clinical problems. Subsequently, it formulates a list of probable differential diagnoses by synthesising information from the available data which aligns with the gathered clinical evidence. Furthermore, the chatbot is directed to provide a detailed series of investigations for the listed differentials, including justifications for each recommended test, ensuring that every diagnostic step is supported by robust clinical rationale. Moving forward, a comprehensive management plan is constructed by the chatbot, incorporating strategies for the prevention and management of complications specific to the clinical situation and medical context identified throughout its analysis. Finally, the chatbot encapsulates its analysis and recommendations in a concise summary.

This structured, step-by-step approach not only enhances the chatbot’s ability to generate clinically relevant and accurate responses but also promotes transparency in its decision-making process, ensuring traceability, comprehensiveness and clarity in the final AI-generated recommendations.

At the end of the prompt, the chatbots will be further instructed to follow the prompt instructions strictly to reinforce the specific model persona for the desired output. [11] Output formats are standardised throughout the prompt chain; where certain AI model(s) does not support table generation, the outputs will be reformatted into lists manually.

### AI chatbots

AI chatbots will be accessed via Poe (Quora, California, U.S.), a third-party subscription-based AI software platform. We will evaluate the responses generated from Claude 2 (Anthropic, California, U.S.), Gemini Pro (Google DeepMind, London, U.K.), GPT-4.0 (OpenAI, California, U.S.), and a custom bot based on GPT-4.0 (cGPT-4).

The custom bot will be created through the “Create bot” function within the Poe interface. GPT-4 is selected as the foundation model for the custom bot. Four widely recognised clinical references will be integrated into the knowledge base of the custom bot, which will include: Török, E., Moran, E. and Cooke, F. (2017) *Oxford Handbook of Infectious Diseases and Microbiology*. Oxford University Press. [12]; Mitchell, R.N., Kumar, V., Abbas A.K. and Aster, J.C. (2016). *Pocket Companion to Robbins & Cotran Pathologic Basis of Disease* (Robbins Pathology). Elsevier. [13]; Sabatine, M.S. (2022) *Pocket Medicine: The Massachusetts General Hospital Handbook of Internal Medicine*. Lippincott Williams & Wilkins. [14] and Gilbert, D.N., Chambers, H.F., Saag, M.S., Pavia, A.T. and Boucher, H.W. (editors) (2022) *The Sanford Guide to Antimicrobial Therapy 2022*. Antimicrobial Therapy, Incorporated. [15] These references aim to provide domain-specific knowledge to inform the generated responses by the custom bot.

The AI chatbot response variability is configured to the pre-determined temperature setting as defined by Poe, which will be most applicable to the general user. Temperature, a hyperparameter in the GenAI model, determines the degree of randomness in its responses. A lower setting produces more predictable responses while a higher setting produces answers with greater variability and creativity. [16] The preset temperature configurations for the AI chatbots are as follow: Claude 2 at 0.5, GPT-4 at 0.35, and the custom bot at 0.35; however, the exact temperature setting for Gemini Pro is not publicly available.

Each clinical scenario will be presented as a new chat using an unconditional prompt to ensure unbiased outputs. All scenarios will be inputted by E.K.Y.C. and processed on a pre-specified date to ensure output consistency.

### Blinding, randomisation and data compilation

The dataset will include 40 unique clinical scenarios, individually processed four AI chatbots (i.e., Claude 2, Gemini Pro, GPT-4.0 and cGPT-4), resulting in 160 total outputs. All study authors (except E.K.Y.C.) and human evaluators will be blinded to the original authors of the clinical scenarios, as well as the identity of GenAI model for the chatbot output.

To ensure objective assessments and minimise evaluator biases, all investigators, except E.K.Y.C, will be blinded to the clinical scenarios and chatbot outputs. Dual-level randomisation will be employed, where the clinical scenarios will be randomised before being inputted into the chatbots, and the corresponding AI-generated responses will be further randomised before being subjected to human evaluation. Randomised clinical scenarios and corresponding AI chatbot outputs will be uploaded onto the Qualtrics survey platform (Qualtrics, Utah, U.S.) for human evaluation and grading. Assigned gradings will be recorded automatically by the survey platform for data compilation and analysis.

### Human evaluation

Two groups of human evaluators will be invited to conduct the study. Human evaluators will be selected from the Department of Microbiology at the University of Hong Kong, the Department of Medicine (Infectious Disease Unit) at QMH, and the Department of Medicine & Geriatrics (Infectious Disease Unit) at Princess Margaret Hospital. The first group will consist of three specialists while the second group will include three resident trainees. These two panels of evaluators will be representative of a diverse range of clinical experience and expertise, therefore offering a broad spectrum of insights into the analytical performance of AI chatbots in the clinical setting.

The evaluators will be presented with the clinical scenarios in random order along with the corresponding AI chatbot-generated responses, which will also be anonymised. Evaluators will be blinded to the identity of AI chatbots during the evaluation process. They will be instructed to read the entire clinical scenario and each of the generated responses in full, before grading. Blinded evaluations will be conducted independently during the evaluation period.

### Evaluation scale

AI chatbot responses will be evaluated systematically using a 5-point Likert scale across four clinically relevant domains: (1) factual consistency, (2) comprehensiveness, (3) coherence and (4) medical harmfulness (Table 1). [6]

**Table 1:** AI chatbot evaluation scale and rubric.

Domains	1	2	3	4	5
Factual consistency	Unverified/ Non-factual	Insufficiently verified facts	Partially verified facts	Predominantly verified facts	Fully verified facts
Comprehensiveness	Limited coverage	Partial coverage	Considerable coverage	Extensive coverage	Complete coverage
Coherence	Wholly incoherent	Substantially incoherent	Moderately incoherent	Minimally incoherent	Fully coherent
Medical harmfulness	Severely harmful	Moderately harmful	Mildly harmful	Minimally harmful	Harmless

Factual consistency will be assessed by examining whether the information synthesised by the AI chatbots are verifiable and factual, pertaining to the clinical data provided in the scenarios. Comprehensiveness will be assessed by the degree to which the generated response encapsulated all the necessary information required to fulfil the objectives specified in the prompt template, ensuring a detailed and thorough analytical assessment. Coherence will be evaluated based on the chatbot’s ability to produce a logically structured and clinically impactful analysis that adhered to the step-by-step guidance of the prompt template. Medical harmfulness will consider the likelihood of the generated output to inflict patient harm, which encompassed recommending inappropriate investigations, suggesting harmful treatments, or offering incorrect management strategies due to misinterpretation or erroneous fabrications (e.g., hallucinations).

### Outcomes

The primary outcome will be the composite score comparisons between AI chatbots. Secondary outcomes will include domain-level comparisons across generated responses, and correlation analysis between composite scores and characteristics of clinical scenarios and AI chatbot output.

## Statistical analysis

### Descriptive statistics

Descriptive statistics will be presented as median (interquartile range, IQR) and mean (standard deviation) values. [17,18] The Shapiro-Wilk test will be employed to assess the normality of the data distributions.

### Internal consistency

The internal consistency of the Likert scale items—factual consistency, comprehensiveness, coherence, and medical harmfulness—will be assessed using Cronbach’s alpha coefficient. This analysis ascertains whether the four domains collectively contribute to a single underlying construct, therefore appropriate for creating a composite score. [17]

### Composite score evaluation

Composite scores (range, 4-20) will be constructed by the summation of the mean scores of all four domains. Differences in mean composite scores among chatbots will be examined using one-way Analysis of Variance (ANOVA). Tukey’s Honest Significant Difference (HSD) test will be applied for post-hoc pairwise comparisons. [18,19] Paired t-tests will be used for within-group comparisons of composite scores between specialist and non-specialist evaluators.

### Domain-level evaluation

At the domain level, Kruskal-Wallis H-test with Bonferroni correction will be used to compare median values across groups. This analysis is conducted for each domain variable to assess differences between AI chatbots. [20] Furthermore, we will evaluate the frequency of responses crossing critical thresholds—such as “insufficiently verified facts” in the factual consistency domain, or “substantially incoherent” in the coherence domain. Prevalence ratios will be computed to compare incidence rates between different generated responses. [21]

### Correlation analysis

Spearman correlation coefficients will be calculated to investigate the relationship between composite scores and word counts from scenario inputs and the corresponding generated outputs. This investigates whether the quantity of text correlates with the quality as perceived through the composite scores.

### Statistical significance

A p-value of less than 0.05 will be considered statistically significant.

## Ethics and dissemination

The study protocol was reviewed and approved by the Institutional Review Board of the University of Hong Kong (HKU)/ Hospital Authority Hong Kong West Cluster (HKWC) – HKU/HA HKW IRB–UW 24-108. Informed consent was exempted.

The data collected in this study will be retrospective in nature, where they will be recorded for clinical purposes. All patient data will be fully de-identified prior to analysis, ensuring that privacy and confidentiality will not be breached. The findings of the study will be published in peer-reviewed academic journals and presented in abstract form at relevant scientific conferences.

### Status and timeline of the study

The study is currently in the evaluation phase, having successfully recruited a qualified panel of clinical microbiologists and infectious disease physicians in January 2024. These evaluators are actively reviewing the provided clinical scenarios. Preliminary analysis will be performed in March 2024. We aim to finalise data analysis by May 2024 and to have a complete report ready for peer review and publication by early 2025.

## Results and discussion

In this protocol, we hypothesise that analytical performance of AI chatbots in real-life clinical scenarios could be objectively measured using a standardised assessment protocol and graded by clinically experienced human evaluators. We also hypothesise that in the evaluation of structured clinical scenarios, AI chatbots primed with domain-specific knowledge in medical sciences could generate clinically relevant recommendations within the boundaries of the prompt template and the scope of the provided clinical data. We further hypothesise that AI chatbots could assist clinicians by providing accurate, comprehensive, and coherent analysis in clinical consultations, without posing medical harm.

In designing this study, we will employ several strategies to limit major biases. Recognising the critical importance of data quality, we will institute a rigorous data curation phase where clinical documents will be reviewed, cleaned, and standardised to ensure AI chatbot operates on high-integrity data. To address the potential for evaluator bias, we will adopt multiple blinding and randomisation procedures, including evaluator blinding, scenario randomisation and response randomisation. Moreover, we will select two diverse groups of evaluators to encompass a broad spectrum of clinical experiences, ensuring our study reflects the varied insights from both specialists and non-specialist doctors.

Nonetheless, there remain several key limitations that bear consideration when interpreting this study. One of the primary limitations is that this study does not accommodate for the inherent potential of continued learning and adaptation by AI chatbots over time. Advances in machine learning suggest that GenAI performances could be improved with continued exposure to clinical scenarios [22], a factor that our current zero-shot prompting protocol does not address. To refine the performance and reliability of LLMs, future research may consider longitudinal study designs or continuous evaluation methods to track the evolution of AI performance with the use of reinforcement learning and human feedback. [23]

Additionally, our protocol will rely on historical clinical data, which will not capture and reflect the dynamic and often unpredictable nature of real-time clinical decision-making. The inherent variability and fluidity of real-life clinical environments are difficult to replicate in a cross-sectional observational study, invariably limiting the generalisability of our findings.

The integrity of chatbot-generated responses is directly tied to the quality of inputted clinical data. [24] Inaccuracies, inconsistencies, or gaps in the original clinical documents may significantly compromise the performance of GenAI models and mask their true capabilities. Advances in natural language processing to automatically extract clinical data from electronic health records may help to mitigate some of these data quality challenges by providing more comprehensive inputs. [25]

Limited number of human evaluators represent another limitation. The study outcomes are dependent on the evaluators’ proficiency and their interpretation of the generated responses. Selected evaluators’ perspectives may not encapsulate the wide-ranging opinions and approaches that exist within the broader medical community, potentially leading to evaluations that do not fully capture the diversity of clinical judgments.

Finally, there are concerns regarding the evaluation scale utilised in this study, which has not been validated and may introduce subjective biases in the evaluation process.

To conclude, this study will represent a significant step towards understanding the analytical potentials of AI chatbots in the clinical setting. While the initial results will provide valuable insights into the capabilities and limitations of AI chatbots in processing and analysing clinical data in a structured manner, the limitations identified must be carefully considered.
